# Characterising ^18^F-fluciclovine uptake in breast cancer through the use of dynamic PET/CT imaging

**DOI:** 10.1038/s41416-021-01623-3

**Published:** 2021-11-18

**Authors:** N. P. Scott, E. J. Teoh, H. Flight, B. E. Jones, J. Niederer, L. Mustata, G. M. MacLean, P. G. Roy, D. D. Remoundos, C. Snell, C. Liu, F. V. Gleeson, A. L. Harris, S. R. Lord, D. R. McGowan

**Affiliations:** 1 Department of Oncology, University of Oxford, Oxford, UK; 2 Oxford University Hospitals NHS Foundation Trust, Oxford, UK; 3 Blue Earth Diagnostics Ltd, Oxford Science Park, Oxford, UK; 4 Royal Berkshire NHS Foundation Trust, Reading, UK; 5 Nuffield Department of Surgical Sciences, University of Oxford, Oxford, UK; 6 Mater Research, University of Queensland, Brisbane, QLD Australia; 7 Mater Pathology, Mater Hospital Brisbane, Brisbane, QLD Australia; 8 Faculty of Medicine, University of Queensland, Brisbane, QLD Australia; 9 MRC Weatherall Institute of Molecular Medicine, Oxford, UK

**Keywords:** Translational research, Cancer imaging, Breast cancer

## Abstract

**Background:**

^18^F-fluciclovine is a synthetic amino acid positron emission tomography (PET) radiotracer that is approved for use in prostate cancer. In this clinical study, we characterised the kinetic model best describing the uptake of ^18^F-fluciclovine in breast cancer and assessed differences in tracer kinetics and static parameters for different breast cancer receptor subtypes and tumour grades.

**Methods:**

Thirty-nine patients with pathologically proven breast cancer underwent 20-min dynamic PET/computed tomography imaging following the administration of ^18^F-fluciclovine. Uptake into primary breast tumours was evaluated using one- and two-tissue reversible compartmental kinetic models and static parameters.

**Results:**

A reversible one-tissue compartment model was shown to best describe tracer uptake in breast cancer. No significant differences were seen in kinetic or static parameters for different tumour receptor subtypes or grades. Kinetic and static parameters showed a good correlation.

**Conclusions:**

^18^F-fluciclovine has potential in the imaging of primary breast cancer, but kinetic analysis may not have additional value over static measures of tracer uptake.

**Clinical Trial Registration:**

NCT03036943.

## Background

Molecular imaging of breast cancer through the use of [^18^F]-fluorodeoxyglucose positron emission tomography (FDG-PET) is commonly used for tumour staging and assessment of therapy response. However, it is limited by its poor differentiation of malignant and benign lesions [1], varied sensitivity and inability to pick up certain histologic subtypes [2]. There is therefore a need for alternative molecular imaging tracers that can address limitations such as these. In addition, new drugs are being developed to target breast cancer metabolism, including amino acid and mitochondrial metabolism, and ways to assess their biological activity and stratify patients are needed [3]. Amino acid uptake is upregulated in breast cancer [4], therefore synthetic amino acid analogues may be a useful tool in molecular imaging of this disease [5]. ^18^F-Fluciclovine (anti-1-amino-3-fluorocyclobutane-1-carboxylic acid or FACBC) is a synthetic amino acid PET radiotracer already licensed for use in patients with biochemically recurrent prostate cancer [6, 7], and its utility for imaging breast cancer is currently under investigation [5, 8, 9].

Quantitative imaging techniques in PET, looking specifically at the distribution of tracer uptake over time, can provide information that reflects the true underlying physiology within the regions of interest [10–12]. Furthermore, using kinetic modelling, quantitative estimates of blood flow into the tumour, ^18^F-fluciclovine transport and intracellular containment can be extracted, as opposed to the commonly used semi-quantitative parameter SUV (standardized uptake value).

We conducted a ‘window of opportunity’ clinical study (FRONTIER) in which we recruited 39 female patients with treatment-naive primary breast cancer. Participants underwent an ^18^F-fluciclovine PET-computed tomography (CT) scan shortly after diagnosis and prior to definitive treatment. The primary objective was to characterise ^18^F-fluciclovine uptake using PET imaging for different breast cancer receptor subtypes. Exploratory objectives were to determine the most suitable kinetic model to describe ^18^F-fluciclovine uptake in patients with invasive breast cancer for the primary tumour and assess differences in tracer kinetics and SUV between tumour receptor subtypes. We also assessed whether there was a relationship between ^18^F-fluciclovine tumour uptake and three surrogate markers of clinical outcome in breast cancer that have previously been shown to correlate with breast tumour FDG uptake: grade, tumour Ki-67 expression and the neutrophil–lymphocyte ratio (NLR) [13, 14]. Ki-67 is a nuclear protein present in all active phases of the cell cycle, except the G0 phase, and is a well-validated measure of breast cancer proliferation, prognosis and response to therapy [15]. We hypothesised that tumours with greater rates of cell proliferation and hence greater anabolic macromolecular requirements would take up ^18^F-fluciclovine to a greater degree. The NLR has been shown to independently associate with increased mortality in breast cancer [16] and correlate with FDG uptake [14]. We hypothesised that tumours with higher NLR would take up ^18^F-fluciclovine to a greater degree.

In preclinical models, metformin has been shown to indirectly stimulate glutamine uptake into tumour cells as a consequence of inhibition of electron transport chain function in tumour mitochondria [17]. Therefore, a comparison of tumour uptake and kinetics in those patients who were and were not taking metformin was also carried out to assess the potential for this tracer to describe the metabolic response to anti-mitochondrial cancer therapies.

## Methods

### Patient selection

Thirty-nine female patients (age > 40 years) with biopsy-proven invasive breast cancer that measured 1.5 cm or more were recruited for the study between March 2017 and October 2018. Exclusion criteria were current pregnancy or breast feeding, multifocal breast cancer, prior treatment for breast cancer and participation in another investigational clinical study within 4 weeks prior to enrolment. Data were also collected describing patient demographics, medical history, tumour size (through ultrasound, mammography or MRI imaging), whether taking metformin, and standard of care breast histology to identify type, grade and receptor status. Receptor status was categorised into three distinct groups: oestrogen receptor positive only (ER+), human epidermal growth factor receptor positive (HER2+) and triple receptor negative (TN). Determination of receptor status was carried out using immunohistochemistry by the Cellular Pathology Laboratory at the John Radcliffe Hospital, Oxford University Hospitals NHS Trust to standardised protocols as part of routine clinical care. Oestrogen receptor Allred score of 2/8 or less was considered negative. For HER2 a score of 3+ on immunohistochemistry was considered positive, and if scored borderline (2+), HER2 in situ hybridisation testing was used as per current UK guidance [18]. The study was prospectively approved by the Oxford A Research Ethics Committee and registered with ClinicalTrials.gov identifier: NCT03036943. All patients involved gave written informed consent.

### PET/CT imaging

Patients were imaged supine with their arms by their side using a Discovery 710 PET/CT scanner (GE Healthcare). They were injected with 370 MBq (±10%) of ^18^F-fluciclovine 30 s into the 20 min dynamic list-mode PET acquisition, which was centred over the breasts. Prior to each PET acquisition, a CT scan was performed for localisation and PET attenuation correction. PET data were reconstructed using a time-of-flight ordered subset expectation maximisation algorithm (VPFX, GE Healthcare) with a standard 6.4 mm Gaussian filter applied post reconstruction. The data were binned into two parallel time sequences, S1 (1 × 30 s, 12 ×  5 s, 6 × 10 s, 5 × 30 s, 10 × 60 s, 1 × 300 s) and S2 (1 × 30 s, 60 × 1 s, 12 × 10 s, 3 × 30 s, 10 × 60 s, 1 × 300 s), and the acquisition was also binned into four 5-min frames. The first 5-min frame will include rapid changes in tracer uptake following injection.

Primary tumours were outlined on the PET/CT images by an experienced radiologist. Cylindrical blood volumes of a diameter of 10 mm were generated within the central part of the descending aorta on at least five consecutive axial PET slices. PET sequences S1 and S2 were then used to produce time–activity curves (TACs) within the volume of interests, representing tracer uptake in the tumours/lymph nodes and blood, respectively. Kinetic analysis was performed on TACs obtained from primary tumours; lymph nodes were excluded due to their size.

### Static analysis

**Fig. 1:**
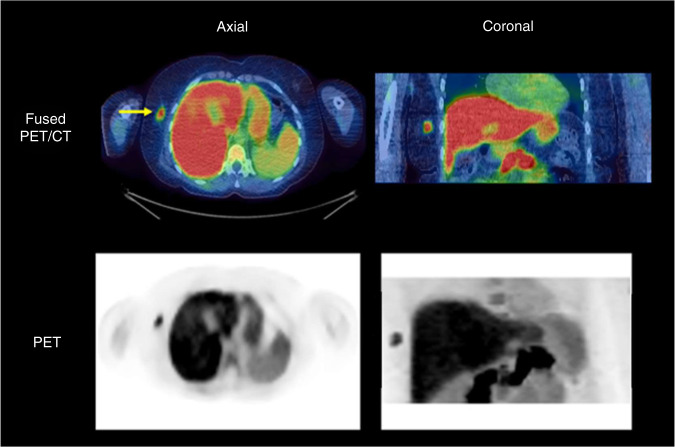
Summed 5- to 10-min dynamic ^18^F-fluciclovine PET and fused PET/CT images of a patient with oestrogen receptor-positive IDC. The primary tumour is indicated by the arrows in the axial plane of the fused PET/CT images.

^18^F-Fluciclovine uptake in tumours was measured for each 5-min time interval. SUV_max_ and SUV_peak_ were calculated using Hermes Hybrid Viewer (Hermes Medical Solutions AB) for each interval, in order to determine which time period demonstrated the highest level of uptake. The time interval in which peak uptake was seen was used for the semi-quantitative measurements of SUV_max_ and SUV_peak_ in primary tumours. The location of the SUV_peak_ volume was independently found for each time interval [19]. Example images of ^18^F-fluciclovine uptake in the time interval 5–10 min post injection are shown in Fig. 1. The liver and pancreas demonstrate high physiological ^18^F-fluciclovine uptake.

### Kinetic analysis

To analyse the dynamic PET data, compartmental modelling was used. This allows the tracer uptake to be mathematically represented via a series of linear compartments; Supplementary Fig. 1 shows diagrams representing one- and two-tissue reversible compartmental models. As ^18^F-fluciclovine uptake is considered to be a reversible process and is not metabolised within cells [6], these were the models that were chosen to fit the time courses of tracer uptake. All kinetic analysis was carried out using PMOD software (version 3.9, PMOD Technologies LLC). Image-derived input functions were obtained using the decay-corrected blood TACs; linear interpolation was then used to fit curves to the data points.

Mathematical models of one- and two-tissue reversible compartmental models (named 1C2K and 2C4K, respectively) were fitted to 38 breast tumour TACs (one TAC was excluded due to patient movement). This was done by minimising the weighted sum of squares between the model fit and the measured TACs to achieve optimised fitting parameters [20]. This was carried out using the Levenberg–Marquardt algorithm

$${{SS}} = \mathop {\sum}\limits_{i = 1}^N {w_i\left( {C_{{PET}}\left( {t_i} \right) - C_{{model}}\left( {t_i} \right)} \right)^2}$$

where $C_{{{{{\mathrm{PET}}}}}}\left( {t_i} \right)$ and $C_{{{{{\mathrm{model}}}}}}\left( {t_i} \right)$ are the imaged and modelled activity concentrations at time $t_i$ (the midpoint of the *i*th time frame) and $w_i$ is the relative weighting factor calculated as

$$w_i = \frac{{\Delta t_ie^{ - \lambda t_i}}}{{C_{{{{{\mathrm{PET}}}}}}\left( {t_i} \right)}}$$

where $\Delta t_i$ is the length of the *i*th frame and *λ* is the decay constant for ^18^F; the methodology is as previously published [13]. The volume of distribution (for the 1C2K model or first compartment for the 2C4K model) was calculated as *K*_1_/*k*_2_.

To ensure global best fits, model fitting was initiated from 200 random starting sets of values. These starting values were suitably constrained (*v*_B_ 0.1–100%, *K*_1_ < 4 mL/min/mL, *k*_2,3,4_ < 4 min^−1^) to ensure appropriate model fitting parameters. The fits were also visually checked within PMOD.

### Assessment of model fit

To determine whether the tracer uptake was adequately described by a particular model, the Wald–Wolfwitz runs test was used. Information criterion testing (Akaike and Bayesian) were also carried out for each model fit, to quantitatively assess how well the model describes the measured data. Further details on these statistical testing methods are described by McGowan et al. [21].

The precision of kinetic parameters from these fits was determined via Monte Carlo simulations: 1C2K and 2C4K model fits to the 38 patient TACs (and corresponding rate constants) were taken as ground truths (one TAC was excluded due to patient movement). A set of 1000 simulated noisy TACs were then generated (as described by McGowan et al. [21] from these ground truth TACs and fitted using both 1C2K and 2C4K models). For each ground truth TAC, average kinetic parameter values were calculated from fits to the 1000 simulated TACs and used alongside the ground truth parameter values to establish individual bias and uncertainty estimates on those values. These individual estimates were then combined to give the overall mean bias (MB) and variance of bias values (*σB*). The mean variance (*σP*) was calculated for each parameter as the average of the parameter variances obtained from the fits to the simulated TACs. *σB* and *σP* values were then combined in quadrature to give an overall uncertainty value *σT*.

$$\sigma {{{T}}} = (\sigma {{{B}}}^2 + \sigma {{{P}}}^2)^{1/2}$$

### Clinical correlations

Tumour specimens were either sampled at the surgery or by ultrasound-guided core biopsy (diagnostic sample, if no suitable material at surgery was available). Following prompt fixation and processing of paraffin blocks, tumours were graded 1, 2 or 3 using the semi-quantitative Nottingham grading system [22] by the Cellular Pathology Laboratory at the John Radcliffe Hospital, Oxford University Hospitals NHS Trust to standardised protocols as part of routine clinical care. Staining for Ki-67 (mouse anti-Ki-67 monoclonal antibody, Dako) was performed on a Leica Bond-max autostainer in the Translational Histopathology Laboratory, Department of Pharmacology, University of Oxford. For Ki-67, the percentage of tumour cells with nuclear staining in at least three separate areas of a section were assessed with a minimum of 100 cells counted in each area and the mean was taken separately by two board-accredited pathologists.

The NLR was derived by simply dividing the absolute neutrophil count by the absolute lymphocyte count from a full blood count taken prior to tumour sample collection (Haematology Laboratory at the John Radcliffe Hospital, Oxford University Hospitals NHS Trust).

### Statistical analysis

Correlations between static SUV parameters and kinetic parameters were assessed and unpaired two-tailed *t* tests were used to compare mean ^18^F-fluciclovine uptake across primary tumours for both SUV and kinetic parameters (*K*_1_, *k*_2_ and the volume of distribution, *V*_d_). Paired two-tailed *t* tests were used to compare the means of tumour SUV_peak_ and SUV_max_ across the four 5-min time intervals. Analysis of variance testing was used to compare means of SUV and kinetic parameters against several clinical parameters, including tumour receptor subtypes, tumour grades and patients taking metformin. Pearson’s correlation coefficient was used to investigate correlations between PET parameters with Ki-67 and NLR. *P* values were considered to be statistically significant if <0.05.

## Results

### Quality of compartment fits to ^18^F-fluciclovine uptake

**Fig. 2:**
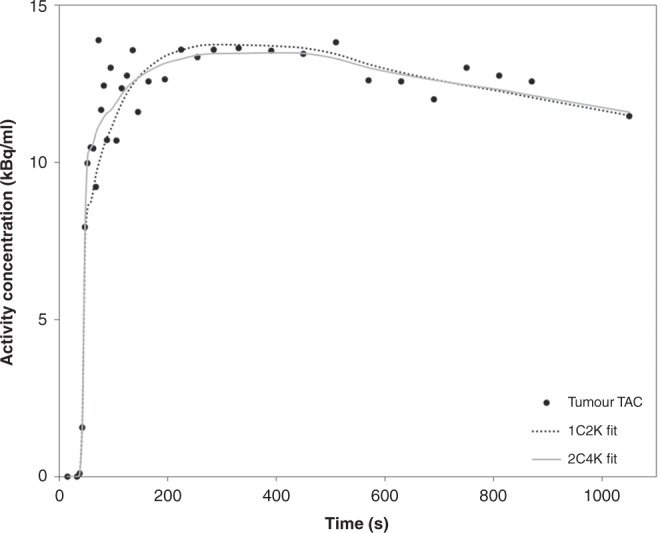
Graphical representation of the time course of tumour tracer uptake in an example patient in the study. One- and two-tissue compartment models have been fitted to the tracer uptake in the tumour.

A detailed breakdown of patient and tumour characteristics are shown in Table 1. Of the eight HER2+ patients, five were ER+ and three ER−. The results from the runs test for the 1C2K and 2C4K compartment models are shown in Table 2. These are shown alongside summed Akaike information criterion (AIC) and Bayesian information criterion (BIC) values for the different models as well as the number of TACs for which each model scored lowest. Fits of the 2C4K model passed the runs test for 31 out of the 38 TACs and had lower total AIC and BIC scores than the 1C2K model. The 1C2K model had an overall higher number of TACs that demonstrated lower AIC and BIC values. Figure 2 shows 1C2K and 2C4K model fits in an example patient TAC and Supplementary Fig. 2 shows the corresponding Logan plot for this patient TAC, which supports the suitability of using a reversible compartment model.

**Table 1:** Patient and tumour characteristics and their corresponding *n* values.

Characteristic	*n* (%)
Recruited patients	39 (100)
Tumour histology	
IDC	39 (100)
Tumour grade	
1	7 (18)
2	12 (31)
3	20 (51)
Receptor subtype	
ER+ HER2−	24 (61.5)
HER2+	8 (20.5)
TN	7 (18)
On metformin	
No	35 (90)
Yes	4 (10)
Tumour diameter (mm)	
Median (range)	24 (15–70)

**Table 2:** Summary of runs-test results and summed AIC and BIC scores for all TACs to which compartmental models were fitted (*n* = 38).

Model	1C2K	2C4K
Runs-test passes		
Runs	23	30
Summed information criteria for all TACs		
AIC	480	245
BIC	662	480
Numbers of TACS for which each model has the best scores		
AIC	21	17
BIC	22	16

Table 3 shows the result of the statistical simulations whereby fits of the 1C2K and 2C4K to measured TACs were used to represent ground truths. The parameter values demonstrate that when 1C2K model fits were used to represent ground truth, 1C2K fits to simulated data had lower MBs and uncertainties than 2C4K fits. For ground truths represented by 2C4K model fits, 1C2K fits to simulated data generally had lower biases and variances than 2C4K fits. Due to the higher accuracy of kinetic parameters in the 1C2K model, this is considered the most appropriate to use to describe ^18^F-fluciclovine uptake for whole tumour TACs.

**Table 3:** Bias and uncertainty results from MC simulations for 1C2K and 2C4K ground truths.

Model fitted	Fitted model parameters					
*Ground-truth 1C2K model*						
1C2K	*v*_B_	*K*_1_	*k*_2_			*K*_1_/*k*_2_
MB	–8%	–3%	–3%			0.3%
*σ*(*B*)	65%	7%	8%			3%
*σ*(*P*)	38%	7%	18%			18%
*σ*(*T*)	75%	9%	20%			18%
2C4K	*v*_B_	*K*_1_	*k*_2_	*k*_3_	*k*_4_	*K*_1_/*k*_2_
MB	–12%	–	–	–	–	–
*σ*(*B*)	66%	–	–	–	–	–
*σ*(*P*)	35%	9%	57%	56%	91%	52%
*σ*(*T*)	75%	–	–	–	–	–
*Ground-truth 2C4K model*						
1C2K	*v*_B_	*K*_1_	*k*_2_			*K*_1_/*k*_2_
MB	64%	–	–			–
*σ*(*B*)	104%	–	–			–
*σ*(*P*)	40%	5%	11%			15%
*σ*(*T*)	111%	–	–			–
2C4K	*v*_B_	*K*_1_	*k*_2_	*k*_3_	*k*_4_	*K*_1_/*k*_2_
MB	21%	–5%	–14%	–36%	–34%	–1%
*σ*(*B*)	76%	13%	142%	170%	80%	36%
*σ*(*P*)	58%	13%	86%	112%	131%	79%
*σ*(*T*)	96%	18%	166%	203%	153%	87%

Values of MB, *σB*, *σP* and *σT* are shown for fitted parameters. *σP* is shown alone when no directly related parameter exists. Values are shown as % of the mean fitted parameter value.

### Correlation of kinetic and static parameters

**Fig. 3:**
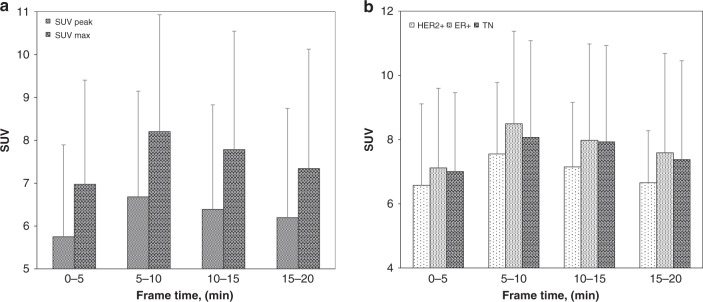
SUVs of ^18^F-fluciclovine in breast tumours at varying time points. **a** SUV_max_ and SUV_peak_ uptake of ^18^F-fluciclovine, **b** SUV_max_ uptake broken down by receptor subtype. Error bars represent the standard deviation from the mean value.

^18^F-Fluciclovine uptake was shown to peak in malignant lesions during the 5–10 min interval post injection; SUV_max_ and SUV_peak_ were significantly higher (*p* < 0.05) during this time frame than any other. The uptake in malignant breast cancer lesions across varying time intervals is graphically depicted in Fig. 3a. When these data were broken down by tumour receptor subtype, the same trend was observed, as shown in Fig. 3b. As a result, any further static analysis was carried out using the uptake in the 5–10 min interval.

**Fig. 4:**
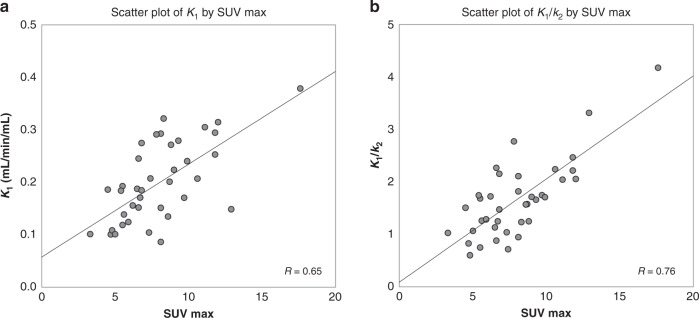
Correlations of SUV_max_ against kinetic parameters for ^18^F-fluciclovine in breast tumours. **a** *K*_1_ and **b**  volume of distribution (*K*_1_/*k*_2_) plotted against SUV_max_. Goodness-of-fit lines are displayed alongside the corresponding goodness of fit value, *R*.

Figure 4 shows the correlation of SUV_max_ against kinetic parameters *K*_1_ and *K*_1_/*k*_2_ in all malignant tumours. Moderate to strong positive correlations were observed for both, with Pearson’s correlation *R* values of 0.65 and 0.76, respectively [23]. The resulting correlation demonstrates good equivalence between static and kinetic parameters for this tracer. In order to study and better understand the underlying differences between them, it was useful to analyse relationships between both static and kinetic values with respect to clinical variables.

### Correlation of kinetic and static parameters with relation to clinical variables

#### SUV and kinetics by receptor subtype

**Fig. 5:**
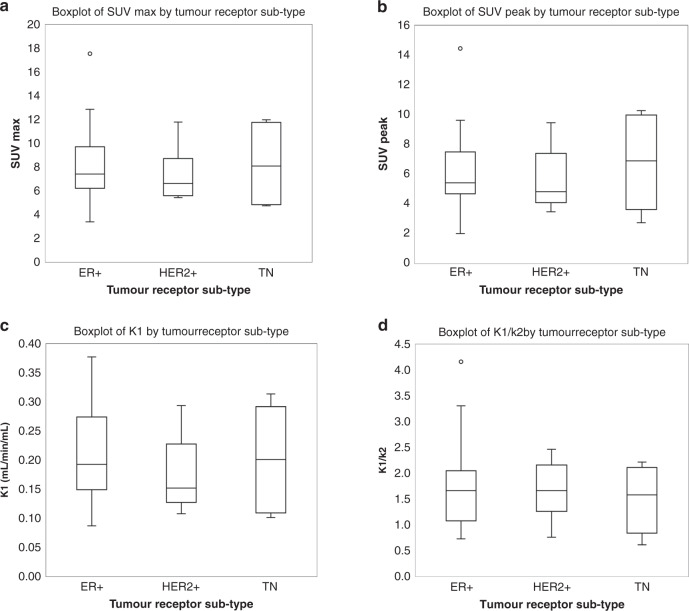
^18^F-fluciclovine uptake across tumour receptor subtypes. **a** SUV_max_, **b** SUV_peak_, **c** K_1_ and **d** volume of distribution plotted for oestrogen receptor positive only (ER+), human epidermal growth factor receptor positive (HER2+) and triple-negative (TN) hormone receptor subtypes.

Tumour receptor status was categorised into three groups: oestrogen receptor positive only (ER+ and HER2−), human epidermal growth factor receptor positive (HER2+) and TN. There were no significant differences in uptake or kinetics between any of the receptor subtypes (Fig. 5).

#### SUV and kinetics by tumour grade, Ki-67 and NLR

Histologic examination of biopsy samples was performed in order to assess the Nottingham grade of each tumour (grade 1, 2 or 3). No significant differences in SUV or kinetics with grade were observed. SUV_max_ values of grade 1, 2 and 3 tumours ranged from 4.2 to 17.6, 3.3 to 12.9 and 4.7 to 12.0, respectively. A trend toward the elevated volume of distribution and SUV_max_ was observed in grade 1 tumours (see Supplementary Fig. 3).

No correlations were seen between the static and kinetic ^18^F-fluciclovine parameters and mean Ki-67 or NLR.

#### SUV and kinetic parameters for patients on and off metformin

**Fig. 6:**
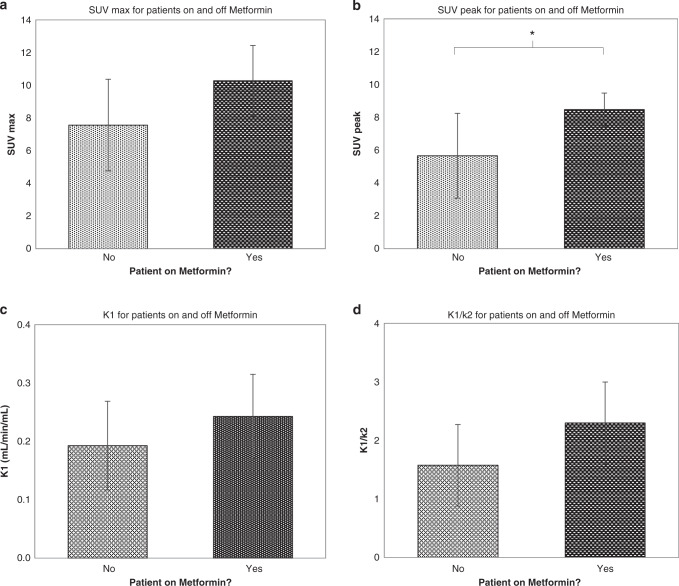
^18^F-fluciclovine uptake in patients taking metformin. Mean values of **a** SUV_max_, **b** SUV_peak_, **c** *K*_1_ and **d** volume of distribution plotted for patients who were on and off metformin. Error bars represent the standard deviation from the mean.

Four study patients (10%) were taking metformin for the treatment of type 2 diabetes. In patients taking metformin, SUV_peak_ was greater (SUV_peak_, 8.5 ± 1.0 versus 5.7 ± 2.6; *p* = 0.04) and a similar trend was observed for SUV_max_ (*p* = 0.07) and the kinetic variables *K*_1_ (*p* = 0.05) and volume of distribution (*p* = 0.06). Plots of static and kinetic parameters for patients on and off metformin are shown in Fig. 6.

## Discussion

In our analysis, kinetic modelling of the dynamic PET images demonstrated that ^18^F-fluciclovine uptake in breast cancer is best described by a reversible one-tissue compartment model. This concurred with the kinetic analysis of ^18^F-fluciclovine uptake in prostate cancer reported by Sorensen et al. in 2013 [6]. The reversible nature of ^18^F-fluciclovine in breast cancer may affect quantitation and lesion detection at later time points after tracer injection. This must be taken into consideration when setting up imaging protocols, where a short uptake time would seem optimal, and also aligns with the approach currently adopted in ^18^F-fluciclovine imaging of recurrent prostate cancer [24, 25]. The peak uptake of this tracer in breast cancer was observed to be at 5–10 min post administration, which is similar to previous studies in both breast [8, 9] and prostate cancer [6]. This suggests that a similar clinical imaging approach to that of recurrent prostate cancer imaging would be appropriate.

Our study showed that there was no association between the surrogate markers of prognosis, Ki-67, grade and NLR. Also, there was no clear evidence that ^18^F-fluciclovine uptake is dependent on tumour receptor status. A slight trend toward an increase in uptake was seen in triple-negative breast cancers compared to other hormone receptor types (not significant), as reported in a smaller study by Tade et al. [8]. A possible explanation is that the transcription factor, Myc, is disproportionally expressed in triple-negative breast cancer and its expression has been associated with tumour cell dependence on glutamine metabolism [26]. In addition, a confounding factor could have been the cellular density. Previous work using mammography has shown differences in cellular density between breast tumour subtypes [27], and tumours with a higher cellular density would have greater PET tracer uptake [28].

Good correlations were seen between the measured static and kinetic parameters within this study and similar trends were observed between tumour characteristics and ^18^F-fluciclovine uptake for both static and kinetic parameters. This would indicate that detailed kinetic analysis for routine clinical purposes does not provide any additional information on the uptake of ^18^F-fluciclovine in breast cancer. However, the kinetic analysis may still have a role in the assessment of subtle differences in ^18^F-fluciclovine uptake, for example, in the use of paired imaging in pharmacodynamic drug trials, a context where kinetic analysis can add value [29].

The uptake of amino acids, in particular glutamine, is an important contributor to the carbon pool necessary for anabolic metabolism and hence cell proliferation. Hence, it was unexpected that no correlation between tumour grade or Ki-67 and uptake of ^18^F-fluciclovine was observed in this study, in contrast to FDG uptake in breast cancer that has shown a positive correlation with grade [30, 31].

We previously showed in a pharmacodynamic ‘window of opportunity’ study that a short course of metformin led to an increase in FDG flux on kinetic analysis of PET-CT when comparing imaging pre- and post treatment [29]. Metformin inhibits complex 1 of the mitochondrial electron transport chain, disrupting the tricarboxylic acid cycle and hence the ability of tumour cells to funnel carbon from glucose toward the synthesis of macromolecules necessary for cell proliferation. In response to mitochondrial dysfunction, tumour cells may switch to amino acids as carbon sources for these anabolic processes [32]. Hence, the observation that tumours had a greater degree of ^18^F-fluciclovine uptake in patients taking metformin was consistent with these preclinical observations. However, this analysis was very much limited by the small number of patients taking metformin and to confirm this finding an appropriately controlled and better powered study in metformin patients would be necessary.

Future work could include investigating the impact of motion correction or improved PET reconstruction algorithms on the breast tumour PET parameters. By utilising these new technologies, it may also enable the assessment of ^18^F-fluciclovine uptake within the lymph nodes.

To our knowledge, this is the largest clinical study to date that assesses the uptake of ^18^F-fluciclovine in primary breast cancer and the first to assess kinetic parameters in this context. This study showed promising results in the use of ^18^F-fluciclovine in the imaging of primary breast cancer across all receptor subtypes and tumour grades. The observation that metformin used by patients was associated with increased uptake of ^18^F-fluciclovine suggests the potential for this tracer to describe the metabolic response to anti-mitochondrial cancer therapies, a number of which are in development.

## Supplementary information

Supp Figure 1

Supp Figure 2

Supp Figure 3

## Data Availability

All datasets used and/or analysed during the current study are available from the corresponding author on reasonable request.

## References

[1] Hyo SL, Yoon W, Tae WC, Jae KK, Jin GP, Heoung KK (2007). FDG PET/CT for the detection and evaluation of breast diseases: usefulness and limitations. Radiographics.

[2] Wahl RL, Siegel BA, Coleman RE, Gatsonis CG (2004). Prospective multicenter study of axillary nodal staging by positron emission tomography in breast cancer: a report of the staging breast cancer with PET study group. J Clin Oncol.

[3] Aroldi F, Lord SR (2020). Window of opportunity clinical trial designs to study cancer metabolism. Br J Cancer.

[4] Plathow C, Weber WA (2008). Tumor cell metabolism imaging. Journal of nuclear medicine: official publication. Soc Nucl Med.

[5] Mcconathy J. ^18^F-Fluciclovine (FACBC) and its potential use for breast cancer imaging. J Nucl Med. 2016;57:1329–30. 10.2967/jnumed.116.175489 27199361

[6] Sörensen J, Owenius R, Lax M, Johansson S (2013). Regional distribution and kinetics of [18F]fluciclovine (anti-[18F]FACBC), a tracer of amino acid transport, in subjects with primary prostate cancer. Eur J Nucl Med Mol Imaging.

[7] Kairemo K, Rasulova N, Partanen K, Joensuu T (2014). Preliminary clinical experience of trans-1-amino-3-(18)F- fluorocyclobutanecarboxylic acid (anti-(18)F-FACBC) PET/CT imaging in prostate cancer patients. Biomed Res Int.

[8] Tade FI, Cohen MA, Styblo TM, Odewole OA, Holbrook AI, Newell MS (2016). Anti-3-^18^F-FACBC (18F-Fluciclovine) PET/CT of breast cancer: an exploratory study. J Nucl Med.

[9] Ulaner GA, Goldman DA, Gönen M, Pham H, Castillo R, Lyashchenko SK (2016). Initial results of a prospective clinical trial of ^18^FFluciclovine PET/CT in newly diagnosed invasive ductal and invasive lobular breast cancers. J Nucl Med.

[10] Muzi M, O’Sullivan F, Mankoff DA, Doot RK, Pierce LA, Kurland BF (2012). Quantitative assessment of dynamic PET imaging data in cancer imaging. Magn Reson Imaging.

[11] Karakatsanis NA, Zhou Y, Lodge MA, Casey ME, Wahl RL, Zaidi H (2015). Generalized whole-body patlak parametric imaging for enhanced quantification in clinical PET. Phys Med Biol.

[12] Dunnwald LK, Doot RK, Specht JM, Gralow JR, Ellis GK, Livingston RB (2011). PET tumor metabolism in locally advanced breast cancer patients undergoing neoadjuvant chemotherapy: value of static versus kinetic measures of fluorodeoxyglucose uptake. Clin Cancer Res.

[13] Cochet A, Pigeonnat S, Khoury B, Vrigneaud JM, Touzery C, Berriolo-Riedinger A (2012). Evaluation of breast tumor blood flow with dynamic first-pass ^18^F-FDG PET/CT: comparison with angiogenesis markers and prognostic factors. J Nucl Med.

[14] Fujii T, Yanai K, Tokuda S, Nakazawa Y, Kurozumi S, Obayashi S (2018). Relationship between FDG uptake and neutrophil/lymphocyte ratio in patients with invasive ductal breast cancer. Anticancer Res.

[15] Dowsett M, Nielsen TO, A’Hern R, Bartlett J, Coombes RC, Cuzick J (2011). Assessment of Ki67 in breast cancer: recommendations from the International Ki67 in Breast Cancer Working Group. J Natl Cancer Inst.

[16] Koh CH, Bhoo-Pathy N, Ng KL, Jabir RS, Tan GH, See MH (2015). Utility of pre-treatment neutrophil-lymphocyte ratio and platelet-lymphocyte ratio as prognostic factors in breast cancer. Br J Cancer.

[17] Fendt SM, Bell EL, Keibler MA, Davidson SM, Wirth GJ, Fiske B (2013). Metformin decreases glucose oxidation and increases the dependency of prostate cancer cells on reductive glutamine metabolism. Cancer Res.

[18] Rakha EA, Pinder SE, Bartlett JMS, Ibrahim M, Starczynski J, Carder PJ (2015). Updated UK recommendations for HER2 assessment in breast cancer. J Clin Pathol.

[19] Wahl RL, Jacene H, Kasamon Y (2009). Lodge M a. From RECIST to PERCIST: Evolving Considerations for PET response criteria in solid tumors. J Nucl Med.

[20] McGowan DR, Skwarski M, Papiez BW, Macpherson RE, Gleeson FV, Schnabel JA (2018). Whole tumor kinetics analysis of ^18^F-fluoromisonidazole dynamic PET scans of non-small cell lung cancer patients, and correlations with perfusion CT blood flow. EJNMMI. Research.

[21] McGowan DR, Macpherson RE, Hackett SL, Liu D, Gleeson FV, McKenna WG (2017). ^18^F-fluoromisonidazole uptake in advanced stage non-small cell lung cancer: a voxel-by-voxel PET kinetics study. Med Phys.

[22] Elston CW, Ellis IO (1991). Pathological prognostic factors in breast cancer. I. The value of histological grade in breast cancer: experience from a large study with long‐term follow‐up. Histopathology.

[23] Ratner B (2009). The correlation coefficient: Its values range between 1/1, or do they. J Target Meas Anal Mark.

[24] Nanni C, Zanoni L, Bach-Gansmo T, Minn H, Willoch F, Bogsrud TV (2020). [18F]Fluciclovine PET/CT: joint EANM and SNMMI procedure guideline for prostate cancer imaging—version 1.0. Eur J Nucl Med Mol Imaging.

[25] Afaq A, Gleeson F, Scarsbrook A, Bradley K, Subesinghe M, MacPherson R (2019). UK guidelines on 18F-fluciclovine PET/CT in prostate cancer imaging. Nucl Med Commun.

[26] Wise DR, Deberardinis RJ, Mancuso A, Sayed N, Zhang XY, Pfeiffer HK (2008). Myc regulates a transcriptional program that stimulates mitochondrial glutaminolysis and leads to glutamine addiction. Proc Natl Acad Sci USA.

[27] Shin HJ, Kim HH, Huh MO, Kim MJ, Yi A, Kim H (2011). Correlation between mammographic and sonographic findings and prognostic factors in patients with node-negative invasive breast cancer. Br J Radiol.

[28] Vranjesevic D, Schiepers C, Silverman DH, Quon A, Villalpando J, Dahlbom M (2003). Relationship between 18F-FDG uptake and breast density in women with normal breast tissue. Journal of nuclear medicine: official publication. Soc Nucl Med.

[29] Lord SR, Cheng WC, Liu D, Gaude E, Haider S, Metcalf T (2018). Integrated pharmacodynamic analysis identifies two metabolic adaption pathways to metformin in breast cancer. Cell Metab.

[30] Basu S, Chen W, Tchou J, Mavi A, Cermik T, Czerniecki B (2008). Comparison of triple-negative and estrogen receptor-positive/progesterone receptor-positive/HER2-negative breast carcinoma using quantitative fluorine-18 fluorodeoxyglucose/positron emission tomography imaging parameters: a potentially useful method for disease characterization. Cancer.

[31] Jo I, Zeon SK, Kim SH, Kim HW, Kang SH, Kwon SY (2015). Correlation of primary tumor FDG uptake with clinicopathologic prognostic factors in invasive ductal carcinoma of the breast. Nucl Med Mol Imaging.

[32] Mullen AR, Wheaton WW, Jin ES, Chen PH, Sullivan LB, Cheng T (2012). Reductive carboxylation supports growth in tumour cells with defective mitochondria. Nature.

