# Supplementary material for: Characterising 18F-fluciclovine uptake in breast cancer through the use of dynamic PET/CT imaging
Source: Br J Cancer. 2021 Nov 18;126(4):598–605. doi: 10.1038/s41416-021-01623-3 (PMC8854436; doi:10.1038/s41416-021-01623-3)
Supplement: Supplementary file 1 — Supp Figure 1 [file 41416_2021_1623_MOESM1_ESM.pdf]

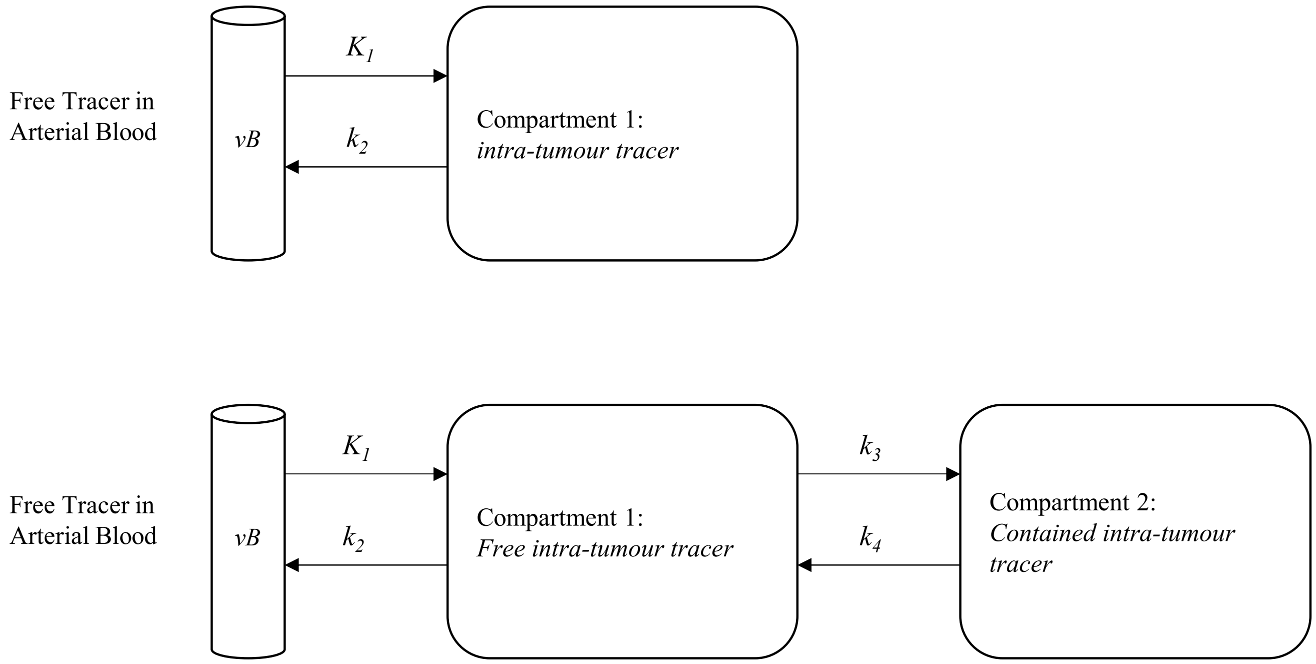

Supplementary Figure 1: Schematic representation of reversible 1-tissue and 2-tissue compartmental models.
