# Supplementary figures and images for: Characterising 18F-fluciclovine uptake in breast cancer through the use of dynamic PET/CT imaging
Source: Br J Cancer. 2021 Nov 18;126(4):598–605. doi: 10.1038/s41416-021-01623-3 (PMC8854436; doi:10.1038/s41416-021-01623-3)

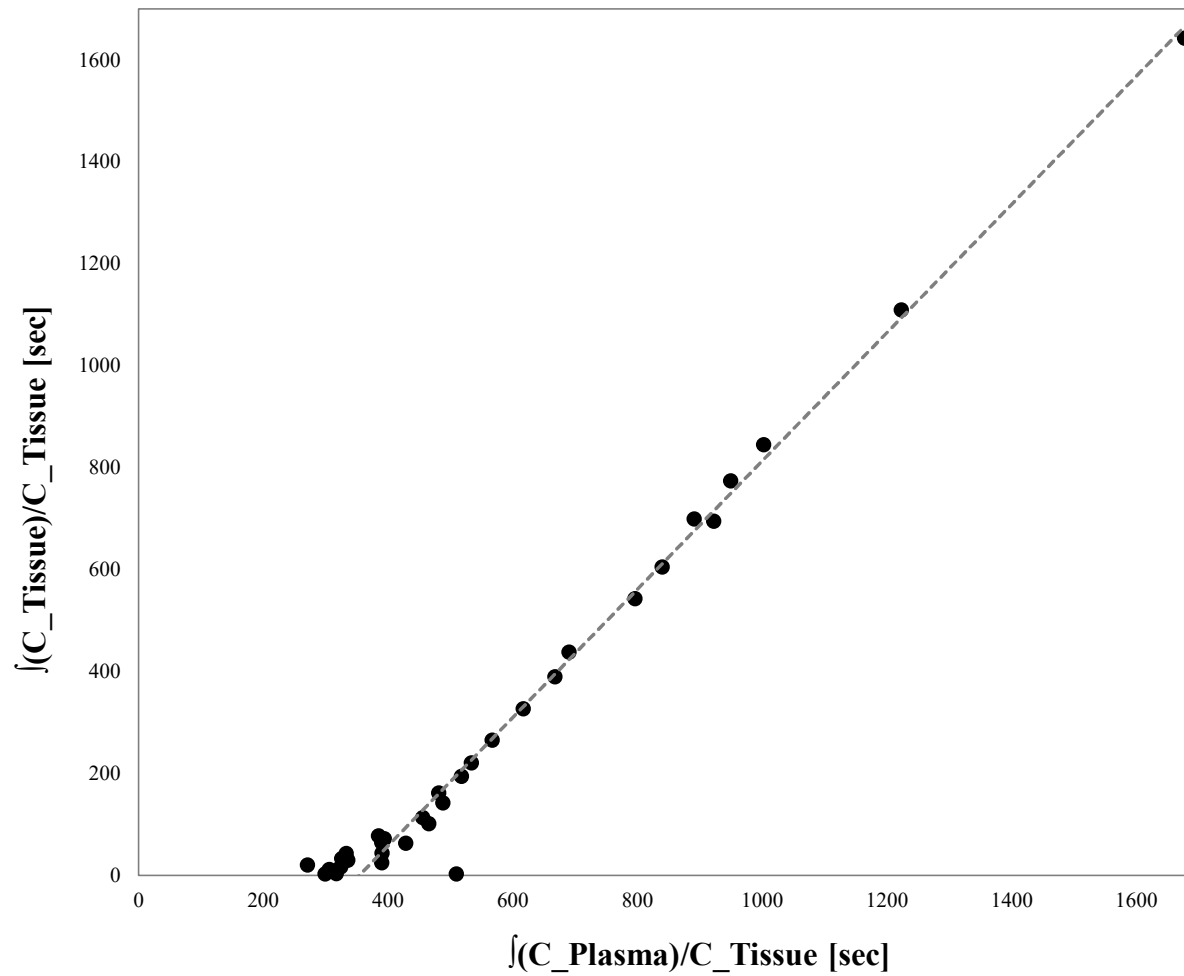

Supplement: Supplementary file 2 — Supp Figure 2 [file 41416_2021_1623_MOESM2_ESM.pdf]
