# Supplementary material for: Characterising 18F-fluciclovine uptake in breast cancer through the use of dynamic PET/CT imaging
Source: Br J Cancer. 2021 Nov 18;126(4):598–605. doi: 10.1038/s41416-021-01623-3 (PMC8854436; doi:10.1038/s41416-021-01623-3)
Supplement: Supplementary file 3 — Supp Figure 3 [file 41416_2021_1623_MOESM3_ESM.pdf]

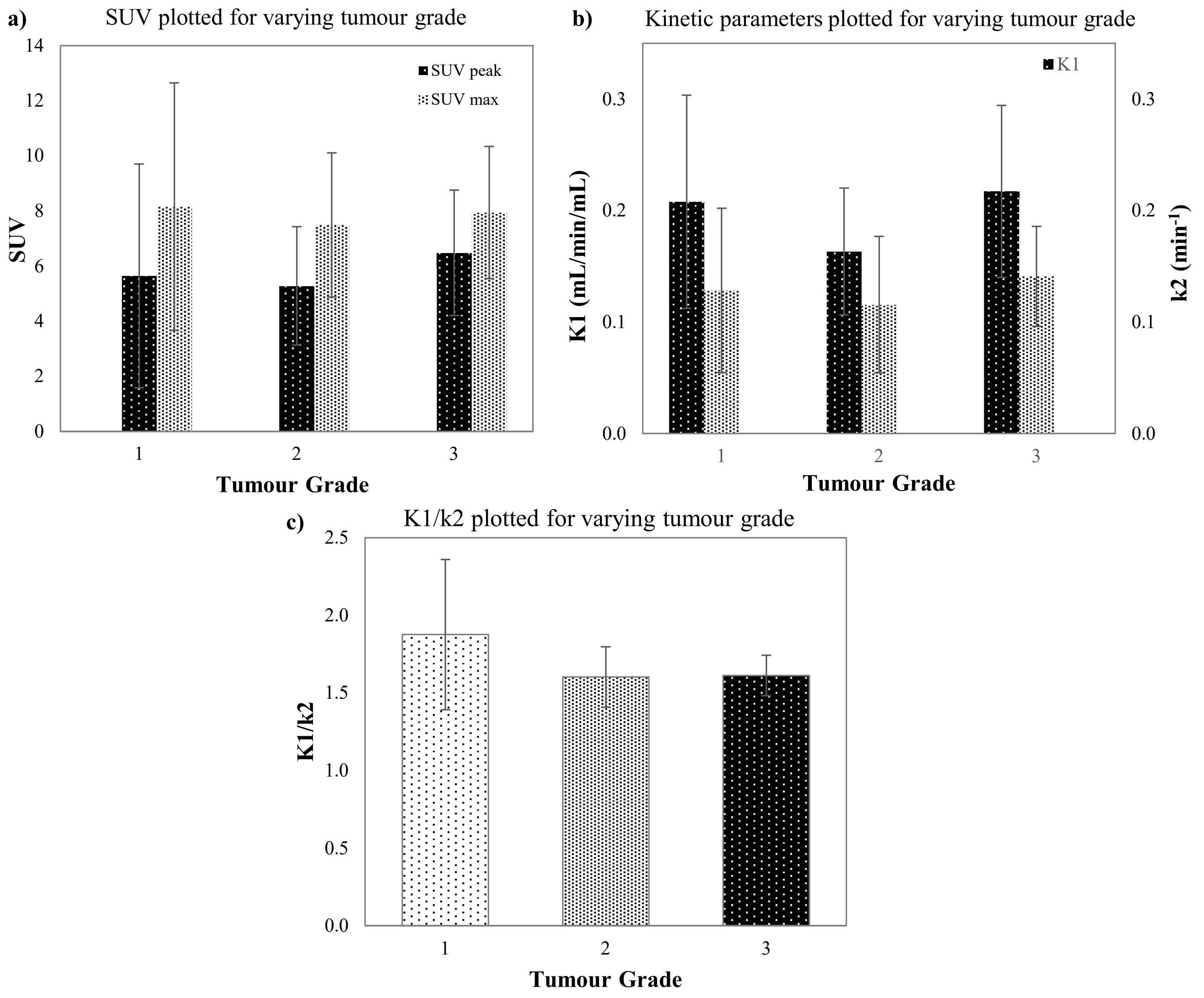

Supplementary Figure 3: a) Mean values of SUVpeak and SUVmax, b) kinetic parameters K1 and k2, and c) volume of distribution plotted for different tumour grades. Error bars represent the standard deviation from the mean.
